# Open Science in Closed Societies

**Published:** 2020-03

**Authors:** Vladimir Mrša

**Affiliations:** Editor-in-Chief of *Food Technology and Biotechnology* and; President of Croatian Association for Scholarly Communication; University of Zagreb, Faculty of Food Technology and Biotechnology, Pierottijeva 6, HR-10000 Zagreb, Croatia

As you can so easily imagine these days, I am writing this editorial from my living room since about half of the human population has been advised not to leave their homes unless absolutely necessary. Our lives have so dramatically changed in the last two months as rarely before. Realising the proportion of the threat imposed by a new virus SARS-CoV2, most European governments reacted by supressing the number of social contacts, closing kindergartens, schools, universities, public transports, most shops, cinemas, theatres, concert halls, all social events, public parks, open markets, and in most countries restricting the movement of their citizens including crossing the local and national borders (*1*). Such massive closing up of our societies has probably never been seen in our history. On the other hand, fighting the challenge of a new pandemic virus requires collaboration and a consorted action of researchers all over the world. Thus, a need for open science has never been as clear as today. It did not take long before the broad human population realized that only science can provide solutions for the ongoing problem. The traditional European scepticism in science and scientists’ opinions (*2*) melted quickly and the trust in the scientific community grew like seldom before. The character of the pandemic we are facing clearly implies that it cannot be fought independently in one country or region but that the global response of health systems throughout the world is required. Under such conditions, the importance of scientific communication needs no particular explication. We need fast and accurate dissemination of scientific information in medical sciences. Scientific journals bear the responsibility for reporting scrupulously reviewed papers, particularly in the area important for coping with the epidemic crisis. Therefore, the flux of information has to be fast, but at the same time thoroughly controlled as human lives depend on it. More efficient communication means better collaboration, faster revealing new effective drugs and vaccines, and eventually thousands of spared lives. The public attention these days is mainly to the medical field and the pressure on medical journals is the highest (*3*) but at the same time it is obvious that we are dealing with a problem much broader than just medical. Pandemic has changed our way of life, our views and opinions. It has so many faces that practically every scientific field needs to respond in one way or another. Psychological help for many is needed, engineers are challenged to increase hospital capacities in short time and build enough medical equipment, economists are facing enormous challenges in the production arrest and an abrupt decrease of both supply and demand, law experts are asked how much our constitutionally granted freedoms can or should be restricted in an effort to minimize social contacts and limit the virus spread. Education processes have been challenged by closing the schools and universities leading to massive introduction of distance learning, and employers are asked to reduce the number of employees physically coming to their working places every day. All these demand new processes, new behaviour and new decisions that have to be based on research that has to be undertaken and then disseminated. Even historians may be asked how people used to deal with contagious diseases in the past when they did not have appropriate medicines or vaccines. Well, we do not have an efficient drug either, and it will take another 12 to 18 months for the first vaccine to become publicly available.

Scientific community has always looked at publishing as something scientists must, but not really like to do. It is by far less interesting than revealing new facts and knowledge. The current situation points out how important it is for the science to act, and to act quickly. Exchange of information has become of utmost importance (*4*). And not only the amount but also the quality of disseminated information is important (*5*). As expected, we are witnessing a sharp increase in the number of papers in the virology area, particularly those dealing with corona viruses. Not all these papers are of high quality. Understandably, scientists who work in this field realize that their work now attracts more attention than ever before and they want to publish more papers in shorter time. Publishers and editors, on the other hand, know that each paper dealing with this phenomenon will increase interest in their journal, and eventually its scientometric parameters. This does not necessarily go hand in hand with the quality of science published that requires scrutiny in checking each and every result obtained in the laboratory, critical discussions among scientists before writing the manuscript, and thorough reviews by editors and reviewers before final publishing. The harm that can be done by publishing insufficiently scrutinized data in a situation like this does not need further elaboration and strongly emphasizes the importance of the editorial work. It also directs attention to some of the ’traditional’ questions like the relation between the private (profit-making) and the public (non-profit) publishers, and the models of financing throughout the scientific communication area. It can be expected that the necessities that have become so obvious during the corona crisis would also catalyse the processes that started before the epidemic and are formulated to a large extent in the Plan S of the European Commission.

Epidemiologists, virologists and immunologists have taught us that the SARS-CoV2 is not a very special virus. It is neither the most contagious, nor the deadliest virus we have faced so far (*6*). However, it has caused an epidemic that has changed our world more than any other in recent history. Scientific publishing will have to conform to new standards to provide fast, easy, reliable and inexpensive access to research data both to the scientific community, and to broader public. The quality of papers and the reliability of published data have to become more important than the quantity in the evaluation of scientists and scientific institutions if we want to maintain public trust in science. Scientometric parameters should be considered wisely and in context to provide meaningful information, and scientists should be placed in an environment in which solving riddles of the world should be done for natural curiosity and practical need to make our world a better place, and not for building curricula needed to preserve their everyday existence. Editors and reviewers should be scientists’ friends, not enemies, and journals should be their tools not their judges. Only in such constellation can science act efficiently in finding answers both to our inborn curiosity and to our needs such as the current fight with a new virus. SARS-CoV2 is not the first virus that endangered our society but, more importantly, it is surely not the last one either. The adaptability of the worldwide scientific community to new challenges will determine our quality of life facing new emerging problems and the efficient scientific communication is a prerequisite for any consorted action at the global level.

*Food Technology and Biotechnology* is, as many other journals, affected by SARS-CoV2. We are experiencing a delay in all of our activities, mostly caused by the fact that some of our collaborators are in self-isolation, isolation or are working at half capacity. Additionally, the latest earthquake that struck Zagreb has affected our homes and our office and tested our resilience even more. Even before that, the changes in the financing model introduced by our funding ministry made a major impact on our finances. Nevertheless, we are trying to maintain the journal’s quality and to select the best papers submitted to our journal. This issue contains papers on various interesting topics, from spectrophotometric fingerprinting used to detect starch origin (*7*), new approach to resolving overlapping peaks obtained by HPLC analysis of phenolics and flavonoids (*8*), microencapsulation of grape juice by freeze drying (*9*), spray drying of fermented juçara pulp (*10*) and of transglutaminase enhanced by ultrasound (*11*), identification of durum wheat cultivars with low cadmium content (*12*), development of *Agrobacterium*-mediated transgenic potato cultivars (*13*), inactivation of *Salmonella enterica* by chlorophyllin-chitosan complex and visible light (*14*), identification of bioactive compounds in *Ipomoea tuba* leaf extract with antiproliferative activity (*15*), effect of high sugar content on fermentation dynamics of wine-related yeast species (*16*), to the production of vinegar from soybean molasses (*17*) and of mozzarella cheese with high moisture content (*18*).

I hope that you will enjoy reading the papers and that we will be able to maintain our position as a diamond open access journal in spite of all obstacles. Until next issue, stay well, be safe and stay at home, as long as needed.
